# Real-Time Implementation of Multiband Spectrum Sensing Using SDR Technology

**DOI:** 10.3390/s21103506

**Published:** 2021-05-18

**Authors:** Yanqueleth Molina-Tenorio, Alfonso Prieto-Guerrero, Rafael Aguilar-Gonzalez

**Affiliations:** Electrical Engineering Department, Metropolitan Autonomous University Iztapalapa, Mexico City 09360, Mexico; yanqueleth@xanum.uam.mx (Y.M.-T.); r.aguilar@xanum.uam.mx (R.A.-G.)

**Keywords:** cognitive radios, multiband spectrum sensing, machine learning, wavelets, software-defined radio

## Abstract

In this work, a novel multiband spectrum sensing technique is implemented in the context of cognitive radios. This technique is based on multiresolution analysis (wavelets), machine learning, and the Higuchi fractal dimension. The theoretical contribution was developed before by the authors; however, it has never been tested in a real-time scenario. Hence, in this work, it is proposed to link several affordable software-defined radios to sense a wide band of the radioelectric spectrum using this technique. Furthermore, in this real-time implementation, the following are proposed: (i) a module for the elimination of impulsive noise, with which the appearance of sudden changes in the signal is reduced through the detail coefficients of the multiresolution analysis, and (ii) the management of different devices through an application that updates the information of each secondary user every 100 ms. The performance of these linked devices was evaluated with encouraging results: 95% probability of success for signal-to-noise ratio (SNR) values greater than 0 dB and just five samples (mean) in error of the edge detection (start and end) for a primary user transmission.

## 1. Introduction

The concept of cognitive radio (CR) consists of a radio with the ability to take advantage of ‘*spectral gaps*’ in a timely manner to continue transmitting [1]. CR has been considered as one of the outstanding solutions for spectrum shortages. CR techniques provide the ability to use or share a spectrum in an opportune manner, as well as operate on the best available channel. In this way, CR technology allows secondary users (*SU*), also called unlicensed users, to determine which parts of the spectrum are available and detect the presence of licensed users or primary users (*PU*). When an *SU* operates in an unauthorized band, the CR selects the best available channel, coordinates its access, and, at the right moment, leaves the channel when a *PU* is detected to avoid interferences [2]. Accordingly, the CR paradigm involves the stages of spectrum sensing, decision, sharing, and mobility [3]. Sensing is the ability to timely detect the start of *PU* transmission in the spectrum space by the *SU*. The decision concerns the *SU*’s ability to select the best available spectrum band. Sharing refers to the coordinated access to the channel selected by the *SU*, and spectrum mobility is the capacity of a CR to vacate the channel when a *PU* is detected [3]. The first stage of CR, i.e., spectrum sensing, is essential to determine the presence of a *PU*.

Spectrum sensing in a CR is a term that implies obtaining the characteristics of the spectrum through multiple dimensions, such as time, space, frequency, and code. These characteristics include modulation type, waveform, bandwidth, and carrier frequency. The concept of CR can be applied to other technologies such as the Internet of things (IoT). In [4], a survey focused on the classification and merits of spectrum sensing and spectrum sharing techniques was presented for this technology fusion. Future wireless communication services will require high performance, which will need, among other things, a larger bandwidth. Nevertheless, in various scenarios, the available spectrum spaces are at noncontiguous frequencies. Due to this, it is necessary to have a broad overview of the *PU* activity. One solution is to consider multiband spectrum detection, i.e., perform the detection of available spectral spaces considering a wide bandwidth [5]. The multiband spectrum sensing (MBSS) problem has recently seen contributions representing great promise for implementing efficient CR. Currently, there are, in the literature, many works related to MBSS techniques. For example, in [6], they proposed an adaptive double-threshold spectrum detection method based on the Markov model for environments with a low SNR. This proposal significantly reduces the power/processing consumption for spectrum detection compared to the traditional single-band scheme. In [7], a framework for resource allocation and multiband cooperative spectrum detection was proposed for IoT in cognitive 5G networks. Here, it was emphasized that allocating an optimal number of channels to be sensed using multiband spectrum sensing can significantly decrease energy consumption in comparison with existing spectrum sensing approaches. Another work that stands out with respect to MBSS is [8]. In this article, the midrange method was used to detect the optimal energy level in the diffuse region, which is the area between the low- and high-energy thresholds, for a dual-threshold energy detector (ED).

The authors of the current paper presented a previous study introducing a novel MBSS technique based on multiresolution analysis (MRA) [9,10], combined with machine learning (ML), for edge detection and with the Higuchi fractal dimension (DFH) [11] as a binary decision rule for distinguishing noise and a possible *PU* transmission. In this work [12], one of the three ML algorithms, used for the classification of the coefficients, was the K-means algorithm. By considering this algorithm, it was possible to obtain, on average, 98% certainty of detecting the beginning and end of a *PU* transmission, for an SNR greater than 0 dB. These results were obtained in a simulated environment on the MATLAB platform. Thus, the previously mentioned technique is now implemented in some easy-access SDR (software-defined radio) devices deployed in a real wireless communication environment. This paper allows appreciating the performance of theoretical contributions in a commercial electronic device based on SDR.

According to the literature, there were some recent and high-impact works in the field of interest related to MBSS and its implementation in a simulated or experimental environment with SDR and USRP (universal software radio peripheral) devices. The authors in [13] proposed a cooperative detection algorithm with a sub-Nyquist sampling approach to design a system with significantly reduced cost and energy consumption. In [14], the critical problem of the hidden terminal was considered (in this case, the hidden *PU*), and a specific spatial distribution of the *SU*_s_ was proposed to counteract this problem. In [15], a compression sampling technique was proposed that can effectively reduce the cost of signal acquisition, whose principal objective is to accurately acquire the signal for detection. In [16], individual and cooperative broadband spectrum detection schemes were proposed to reduce power consumption in signal acquisition, processing, and transmission. In [17], an algorithm based on a low-speed multichannel architecture was proposed to detect frequency edges using wavelets in a multiband context for CR networks. In [18], the authors analyzed the detection capacity and developed a self-configured system with dynamic intelligence networks without causing any interference to the *PU*. In this work, two spectrum detection techniques were also mentioned during quantitative analysis: energy detection and band-limited white-noise detection.

Some important contributions related to improving spectrum efficiency over TV with spaces (TVWS) by considering low-cost devices and reduced computational complexity have been made. A good approach to this topic appears in [19]; here, the authors used an affordable prototype to sense TVWS. The results showed that the portable prototype was capable of detecting unoccupied frequency bands from 500 MHz to 698 MHz in urban areas. Moreover, in [20], a prototype able to detect up to 10 TV channels was presented. The sensing device was examined in a real environment, and a spectrum occupancy of 30.25% was calculated. In [21], an energy detection model was examined, combined with noise and primary user activity. This proposal was tested in a low-cost open-source sensing station. The proposed model detected TVWS with 9.6% more accuracy than current models. In [22], SDR and USRP devices were used to implement a communication system to detect interferences performing significantly better than the ED in the detection of weak interference, offering the capability of detecting 8 to 10 dB lower values of interference. In [23], experimental results of the USRP hardware implementation for the detection of ‘*gaps*’ in the spectrum were presented with the advantage of requiring fewer samples than the ED and obtaining the same performance. In [24], a new SDR platform architecture was proposed that implements different modulation techniques, and this architecture showed favorable gains with an advantage over traditional techniques, such as spatial multiplexing multiple-input multiple-output (MIMO) systems. In [25], a distributed CR network architecture was presented, in which each CR knows local network state information and performs spectrum sensing, channel estimation for joint routing, and channel access subject to realistic network topology and physical channel effects. The authors in [26] presented a CR system in a real environment in which nodes can either communicate with other nodes via (i) a direct communication with the base station at the macrocell, which helps to enhance network performance, or (ii) device to device that improves spectrum efficiency, whereby traffic is offloaded from macrocell to small cells, using two USRP platforms combined with the GNU radio software toolkit. In [27], the authors used USRP with Raspberry to develop a distributed wireless network in which nodes can communicate with other nodes independently and these can take decisions autonomously. In [28], a small-scale testbed was proposed for dynamic spectrum access in a CR network to find a common channel for communication between two devices (peer to peer, using USRP). A technique for dynamic spectrum access was also presented. In [29], a didactic spectrum analyzer application with multiple functions was presented that uses an SDR device at a very low cost.

Many important contributions related to CR have been implemented considering USRP devices. Nevertheless, in this work, the main motivation consists of showing that SDR devices can be useful for providing information about how a theoretical contribution could behave implemented in real time without a considerable investment compared to the high economic cost that could represent USRP devices [30,31].

This work is organized as follows: in Section 2 a brief description of the considered SDR devices is presented. In Section 3, the implementation of the MBSS technique using SDR technology is explained. Section 4 presents the implemented scenario and results. Section 5 provides the conclusion and a brief discussion.

## 2. Software-Defined Radios

During the last few decades, telecommunications have been in constant evolution. Among the most relevant technological contributions is the digital processor. This item offers to radio equipment the flexibility of a programmable system. In other words, the behavior of a communication system can be modified simply by changing its software. This benefit introduced a new radio paradigm called software-defined radio. Under this paradigm, the task of configuring the behavior of the radio is transferred to the software, leaving the hardware only to implement the radiofrequency front end. With this, the radio is no longer static, it is defined by its circuits and becomes a dynamic element, which can change its operational characteristics, such as bandwidth, modulation, and coding rate, even modified during the execution time according to the software configuration [32].

SDR is defined as “radio in which some or all physical layer functions are defined by software” [32]. In SDR devices, the software modules are executed in real time on microprocessor platforms or digital signal processors. Moreover, most of the devices consider FPGA for transmitting or receiving radio signals. The main operational characteristics of SDRs can be modified at runtime; the system can be easily reconfigured to perform different functions as needed. Due to this, transmitters (Tx) or receivers (Rx) can be created for any type of signal through software or firmware functions [26,27].

SDRs can be used in various radiofrequency technologies, such as Bluetooth, WLAN, GPS, Radar, WiMAX, or LTE. The rapid evolution of wireless communication systems and their standards has made base station software updates a more attractive solution than costly base station replacement, both for the military sector and for the commercial sector. For example, updating the software of the satellite communication equipment deployed will allow changes in communication functionality and multiple uses during the life of the satellite [33]. SDR opens a range of possibilities by making existing types of radio applications easier to implement and by enabling new types of applications. In particular, the computing power and flexibility of the SDR can be leveraged to develop CR [34].

The emergence of relatively affordable devices that receive and digitize radiofrequency signals has brought SDR to the desks of many communications engineers. Nevertheless, the latest availability of very-low-cost SDR devices, such as RTL-SDR, has brought SDR to the home desks of undergraduate and graduate students, as well as professional engineers and creator communities [35]. In this work, the implementation of an MBSS technique is proposed with the use of general-purpose communication devices such as SDR-RTL [36], HackRF One [37], and LimeSDR Mini [38]. Table 1 shows the main characteristics of these devices.

## 3. Implementation of MBSS Technique Using SDR Technology

**Figure 1:**
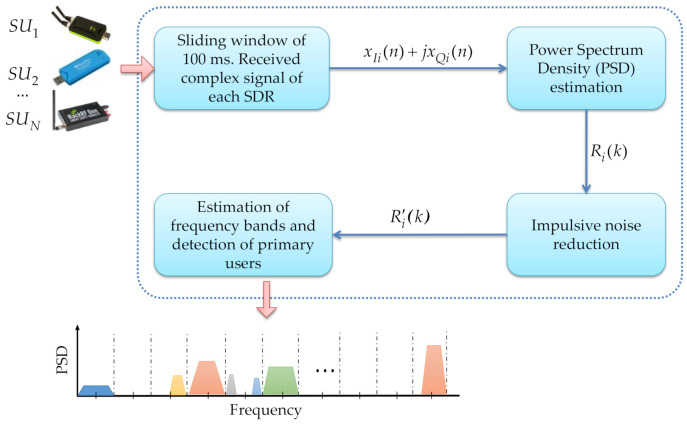
General description of implemented MBSS scenario.

One of the main motivations of the work, previously developed by the authors [12], is to propose a MBSS technique that is both (i) adequate for correctly detecting a primary user and (ii) having a computational complexity allowing real-time implementation in a wireless communications environment. Low-cost devices easy to install and program, such as the SDR devices explained above, are considered. Hence, Figure 1 shows the general scenario for the MBSS using low-cost SDR devices to know the occupation of a wide spectrum interval.

In general, the implemented scenario consists of four big blocks: a sliding time window, an estimator of the power spectrum density, a block for reduction in impulsive noise, and a block to determine frequency edges and detection of PUs. Basically, each device collects information from the environment (i.e., each one working as an *SU*), and this information is processed by the estimator of the power spectral density (PSD) to get the spectrum in different bands. Thereafter, this PSD is modified to eliminate noise, especially impulsive noise, through an MRA (discrete wavelets). Lastly, the last block allows deciding if a *PU* is present or not using discrete wavelets, machine learning, and the Higuchi fractal dimension. Next, each block is described in detail.

### 3.1. Sliding Time Window

**Figure 2:**
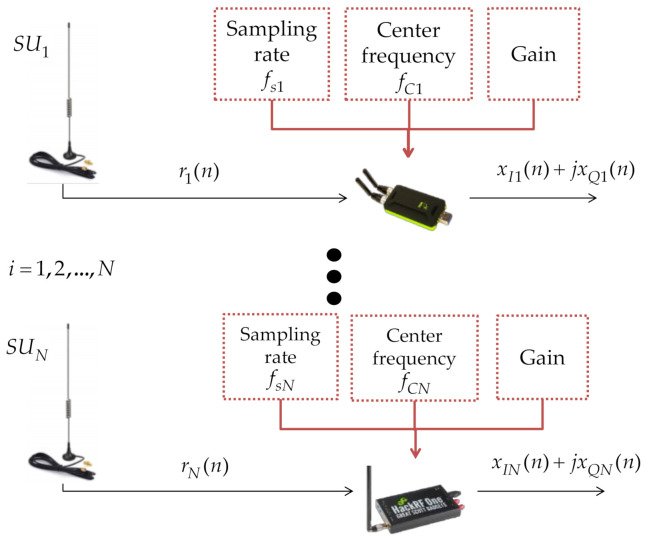
Sensing stage: received signals for the SDR devices, updated every 100 ms.

The block corresponding to the sliding time window is detailed in Figure 2. In this block, the signals received by the SDR devices are conditioned, updating the perception every 100 ms. Each SDR device delivers a complex signal $r_{i}(n)=x_{Ii}(n)+jx_{Qi}(n)$ for i=1,2,…,N for the N devices sequentially connected.

Basically, for each considered SDR device, three parameters are set: the sampling rate $f_{si}$, the center frequency (of reception), and the gain. For the devices used in the proposed scenario, we have a sampling rate of 20 MHz for the HackRF One, 3.2 MHz for the RTL-SDR, and 30.72 MHz for the LimeSDR. The initial gain of each device is 0 dB, which means that the power of the signal perceived by each device is not increased. Nevertheless, this parameter can be modified if the *PU* transmission power is imperceptible by the SDR device (this value is chosen by the user).

**Figure 3:**
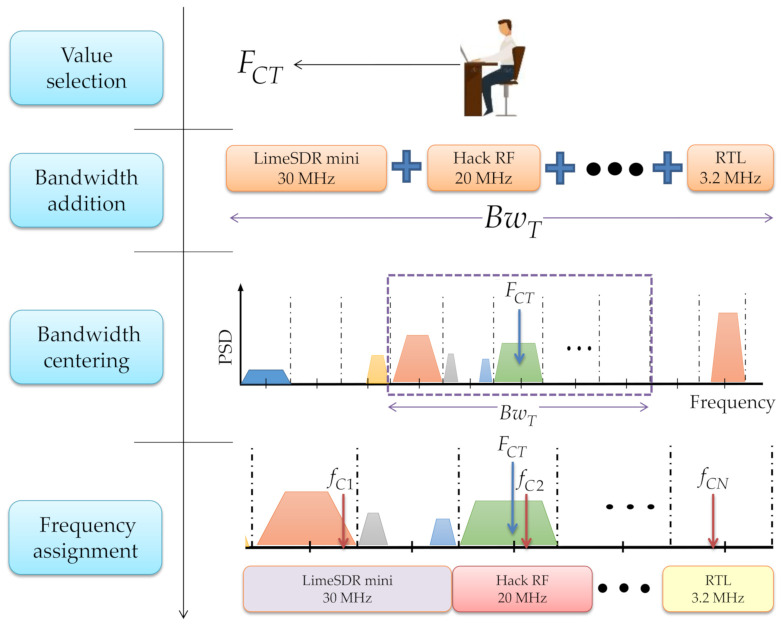
Carrier frequency assignment of each connected device to conform the observed multiband spectrum.

The central frequency $F_{CT}$ of desired spectrum (multiband spectrum bandwidth) is chosen by the user according to the following steps (Figure 3):

- The bandwidth of each connected SDR device is added to conform the complete bandwidth $Bw_{T}$ to be observed.
- The total bandwidth $Bw_{T}$ is centered in $F_{CT}$.
- Lastly, the center frequency $f_{Ci}$ of each connected device is assigned.
- Steps are repeated every time the value of $F_{CT}$ is changed.

### 3.2. Power Spectrum Density Estimation

From the received signal $r_{i}(n)=x_{Ii}(n)+jx_{Qi}(n)$, which is a complex signal that belongs to the time domain, the PSD is obtained on a discrete linear scale of the frequency $R_{i}(k)$ through the Welch estimator [39] for each connected device every 100 ms (frame time).

Welch’s method [40] (also called the periodogram method) for estimating power spectra is carried out by dividing every signal frame of 100 ms into successive blocks or windows, estimating the periodogram (i.e., the squared magnitude of the fast Fourier transform (FFT)) for each block, and averaging over the total number of windows forming each frame. The m-th windowed, zero-padded block is considered from the signal $r_{i}(n)$ denoted by

(1) $$r_{i,m}(n)\;\;=\;\;\omega (n)\;r_{i}(n+mh),n=0,1,\ldots ,M-1,\;\;m=0,1,\ldots ,K-1,\;$$

where h is defined as the window hop size and *K* denotes the number of available windows. Then, the periodogram of the m-th window is given by

(2) $$P_{r_{i,m}}(k)=\frac{1}{M}{\left|FFT(r_{i,m})\right|}^{2}=\frac{1}{M}{\left|\sum_{n=0}^{M-1}r_{i,m}(n)e^{-j2\pi nk/M}\right|}^{2}\;k=0,1,\ldots ,M-1,$$

and the Welch estimate of the power spectral density is given by

(3) $$R_{i}(k)=\frac{1}{K}\sum_{m=0}^{K-1}P_{r_{i,m}}(k)\;\;k=0,1,\ldots ,M-1.$$

**Figure 4:**
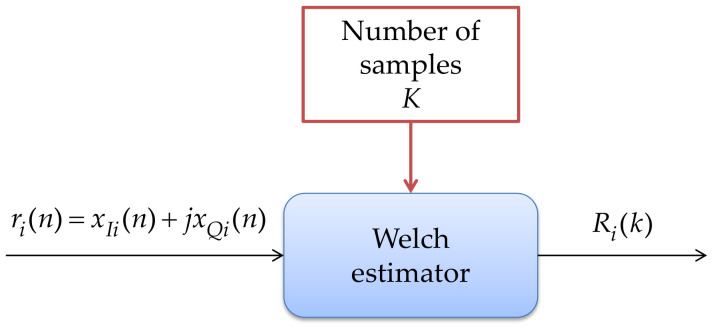
PSD estimation via the Welch method.

In other words, it is just an average of periodograms across time over each frame of *P* points in 100 ms. When ω(n) is the rectangular window, the periodograms are formed from nonoverlapping successive blocks of data. For other window types, the analysis frames typically overlap. In this implementation, a 50% overlapped Hamming window is used and the number of *M* samples contained in $R_{i}(k)$ is chosen according to the device: 512, 1024, 2048, or 4096 samples for the RTL-SDR. For the cases of HackRf One and the LimeSDR Mini, the number of samples can be 1024, 2048, 4096, or 8192. These essential data represent a compromise in the real-time implementation between having many samples to locate the *PU* with great precision or choosing few samples sacrificing the precision to locate the transmission of the *PU* to reduce the execution time and the assignment of computational resources. The structure and operation of this block are shown in Figure 4.

### 3.3. Impulsive Noise Reduction

Due to the nature of the devices used in this work, the addition of a classical noise (flat spectrum) is disturbed by the apparition of spikes in the PSD estimation. These spikes are called impulsive noise and, in general, are more significant with the SDR devices than those observed by a commercial spectrum analyzer. Eliminating this type of noise is necessary to improve the results obtained by the proposed MBSS technique. Many methods have been proposed to mitigate this noise, even novel techniques such as compressed sensing [41] or a recursive least square method based on the state space variant [42].

**Figure 5:**
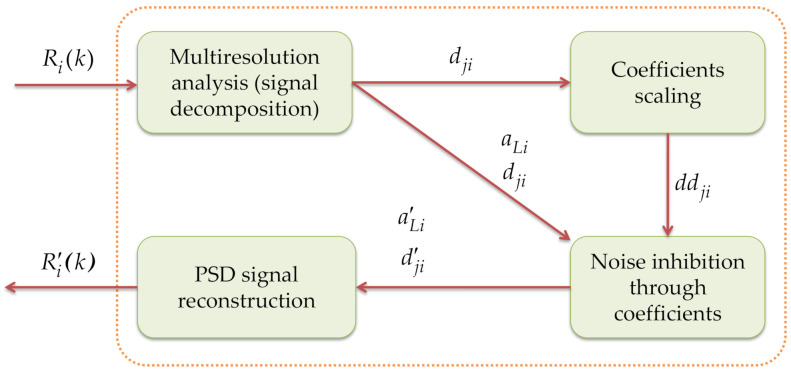
Operation of the impulse noise reduction module.

In this research work, an impulsive noise elimination module is proposed, which is undoubtedly a novel proposal to mitigate abrupt noise changes in the PSD signal that is processed by the MBSS technique. The operation of this module is shown in Figure 5. The elimination of impulsive noise along every frame is done through the approximation and detail coefficients issued from an MRA [9] acting over the PSD estimate.

In the next sections, the operation of each submodule that shapes the impulsive noise elimination module is described in detail.

#### 3.3.1. Multiresolution Analysis: PSD Decomposition

First, to the $R_{i}\left(k\right)$ signal (PSD in a linear scale, the result of applying the Welch estimator), the MRA is sequentially applied to i-th device, resulting in the approximation coefficients $a_{Li}$ at decomposition level L and the detail coefficients $d_{ji}$ at levels going from j=1,2,…,L, as given by Equation (4) [9].

(4) $$R_{i}\left(k\right)=a_{Li}+\sum_{j=1}^{L}d_{ji}.$$

**Figure 6:**
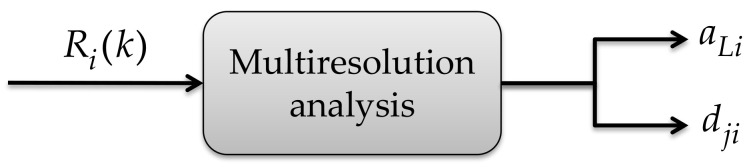
PSD decomposition through the MRA.

The detail coefficients correspond to the discrete wavelet transform of $R_{i}\left(k\right)$ in a dyadic scale. It is well known that the detail coefficients of a signal decomposition, via the MRA, keep the information about singularities (abrupt changes in the signal), which is the case concerning impulsive noise. Because of that, the proposal is based on modifying these detail coefficients to alleviate this problem generated by the impulsive noise. For this, the proposal is only considered at level L = 1, i.e., the approximation coefficients $a_{Li}$ and the detail coefficients $d_{ji}$. The constructed wavelet space from the MRA is the wavelet ‘Haar’. This is shown in Figure 6.

#### 3.3.2. Coefficient Scaling

**Figure 7:**
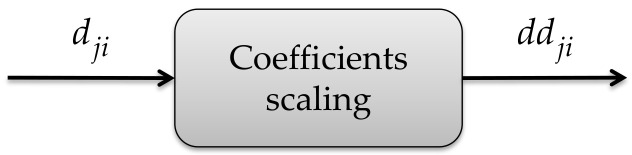
Detail coefficient scaling submodule.

Subsequently, a scaling is made to the detail coefficients $d_{ji}$ of each device i. In this process, 0 is assigned to the coefficient with the smallest value, 1 is assigned to the coefficient with the largest value, and a value between 0 and 1 is assigned to the remaining coefficients $d_{ji}$ as a function of its original value, resulting in the $dd_{ji}$ signal, as it is shown in Figure 7.

#### 3.3.3. Noise Inhibition through Coefficients

**Figure 8:**
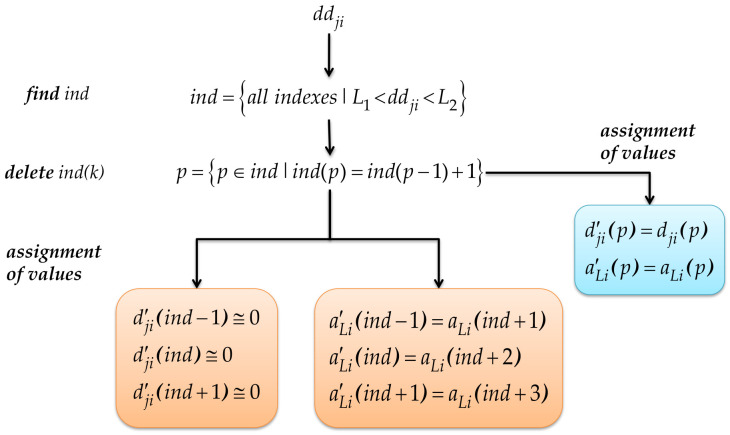
Operation of the noise inhibition submodule.

To reduce high-frequency noise, principally abrupt changes (impulsive noise), in the PSD, the values of the detail and approximation coefficients are reassigned. This task is the most important of this block of noise reduction. The flow chart that describes this submodule is shown in Figure 8.

Once the detail coefficients $dd_{ji}$ have been scaled for each device i, a double-threshold condition, $L_{1}<dd_{ji}<L_{2}|L_{1}=0.01,\;\;L_{2}=0.4$, is applied to the scaled coefficients $dd_{ji}$, and these indices must not be consecutive. The indices that satisfy both conditions are stored in the variable ind.

The detail coefficients $dd_{ji}$ of the ind localities and those that are around, previous, and next, are assigned the value of 1 × $10^{-15}$. Practically, the detail coefficients are turning off, such that these components do not affect the next signal to be reconstructed, which is where high-frequency noise exists. In the remaining localities of $d_{ji}^{\prime }$, the original value of $d_{ji}$ is assigned.

With the previous processing, the ind indices of the detail coefficients coincide with the indices of the approximation coefficients, which also have to be modified to remove some abrupt changes when reconstructing the signal; this coincidence is due to the fact that the level of decomposition is L=1. To modify the approximation coefficients $a_{Li}^{\prime }$, a close colony of three samples of approximation coefficients is replicated in the ind indices mentioned. In the remaining localities of $a_{Li}^{\prime }$ the original value of $a_{Li}$ is assigned.

**Figure 9:**
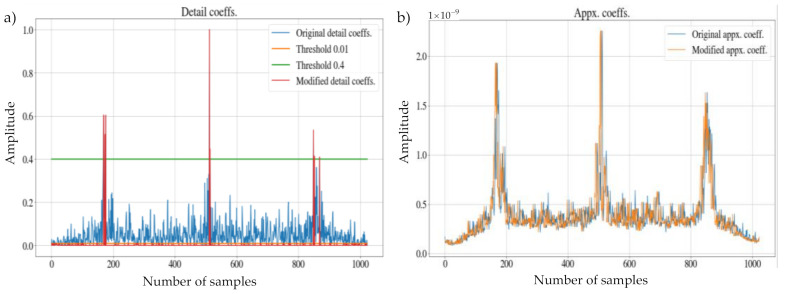
Original and modified coefficients: (**a**) approximation; (**b**) detailed view.

The result of this block corresponds to the approximation coefficients $a_{Li}^{\prime }$ and the detail coefficients $d_{ji}^{\prime }$ with the corresponding attenuations to eliminate the different noise variants mentioned above. Figure 9 shows an example of the difference between the original coefficients and those modified by this block.

#### 3.3.4. PSD Signal Reconstruction

**Figure 10:**
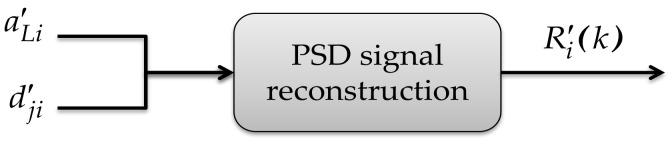
PSD reconstruction after impulsive denoising.

In this block, the new coefficients are used as an input parameter. With them, a wavelet reconstruction is done (again using ‘Haar’). The result of the reconstruction is the signal $R_{i}^{\prime }\left(k\right)$, which is the PSD with the attenuation of the different noise variants on a linear scale. This processing can be seen in Figure 10.

**Figure 11:**
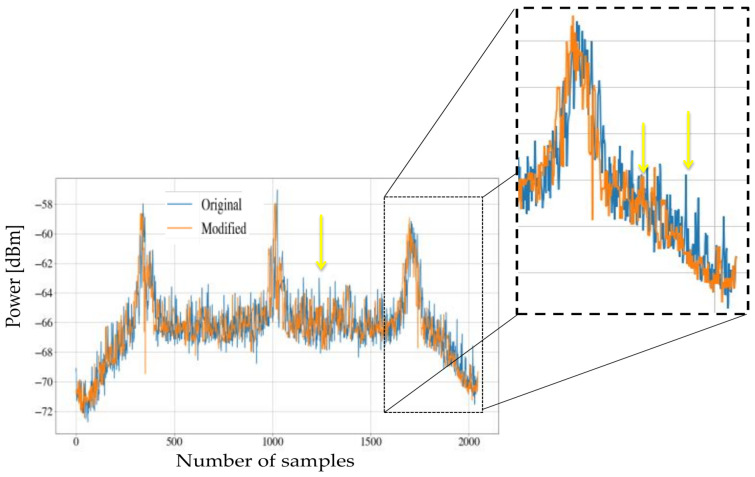
Difference between original PSD and modified PSD.

Taking the previous example, the result of applying the wavelet reconstruction submodule is shown in Figure 11 on a logarithmic scale. In this figure, it is possible to distinguish the changes between the original PSD and the PSD modified by the noise inhibition module.

**Figure 12:**
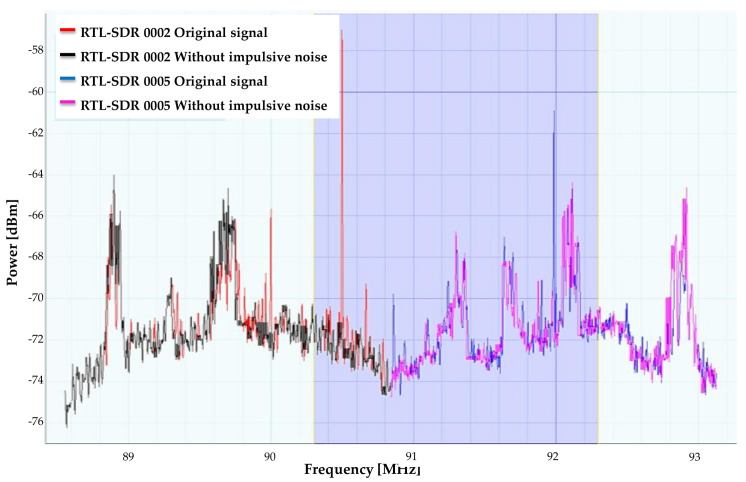
Difference between original PSD and modified PSD using multiple SDR devices.

Another example is shown in Figure 12. This same difference is observed here using two RTL-SDR devices with $F_{CT}=90.8$ MHz, a band exclusively used for broadcast radio [43]. Furthermore, it is possible to analyze a larger frequency range based on the number of connected SDR devices.

In the examples shown in Figure 11 and Figure 12, there are different PSDs that show two challenges. In Figure 11, an abundance of high-frequency noise with very sudden changes is shown, and Figure 12 exhibits an abundance of impulsive noise; in both cases, the noise inhibitor works correctly.

#### 3.3.5. Impulsive Noise Reduction Algorithm

This section presents the pseudocode used for the impulsive noise reduction block.

1. Function noise_reduction ($R_{i}(k)$):
2. $\;\;L_{1}=0.01$
3. $\;\;L_{2}=0.4$
4. $\;\;a_{Li}$; $d_{ji}$ = ***pywt.wavedec*** ($R_{i}(k)$, *‘db1’*, L=1)
5. $\;\;a_{Li}^{\prime }\leftarrow$ $a_{Li}$
6. $\;\;d_{ji}^{\prime }\leftarrow$ $d_{ji}$
7. $\;\;dd_{ji}$ = ***reescale*** ($d_{ji}$)
8.   ind = ***find*** ($d_{ji}>L_{1}$ & $d_{ji}>L_{2}$)
9.     **for** p **in range** (**len**(ind)-1,2,−1):
10.        *aux* =ind*[p−1]*
11.        ***if*** ind*[p] == aux + 1*
12.      ind = ***delete*** (ind,*[p−1])*
13. $\;\;d_{ji}^{\prime }(ind-1)=1\times 10^{-15}$
14. $\;\;d_{ji}^{\prime }(ind)=1\times 10^{-15}$
15. $\;\;d_{ji}^{\prime }(ind+1)=1\times 10^{-15}$
16. $\;\;a_{Li}^{\prime }(ind-1)=a_{L}(ind+1)$
17. $\;\;a_{Li}^{\prime }(ind)=a_{L}(ind+2)$
18. $\;\;a_{Li}^{\prime }(ind+1)=a_{L}(ind+3)$
19. $\;\;R_{i}^{\prime }(k)$= ***pywt.waverec*** ($a_{Li}^{\prime }$; $d_{ji}^{\prime }$, *‘db1’,‘symmetric’)*
20. ***return*** ( ${R^{\prime }}_{i}(k)$ )

### 3.4. Detection of Primary Users

**Figure 13:**
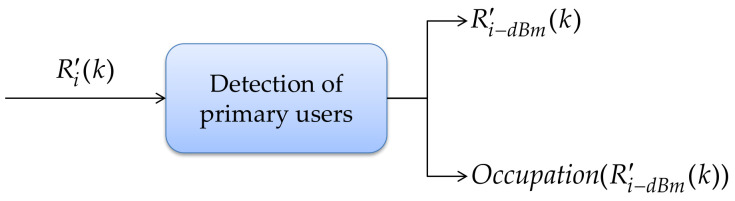
Detection of *PU* module.

This section presents the last block of this implementation: the detection of *PU*_s_ in the spectrum. This procedure receives the PSD on a linear scale $R_{i}^{\prime }(k)$ as an input parameter and delivering two outputs: (i) the signal $R_{i-dBm}^{\prime }(k)$, which is the PSD on the logarithmic scale, and its occupation (i.e., starts and ends of frequency edges of detected bands); (ii) the binary decision, i.e., whether each of these detected bands corresponds to noise or a possible *PU* transmission. This structure is shown in Figure 13.

**Figure 14:**
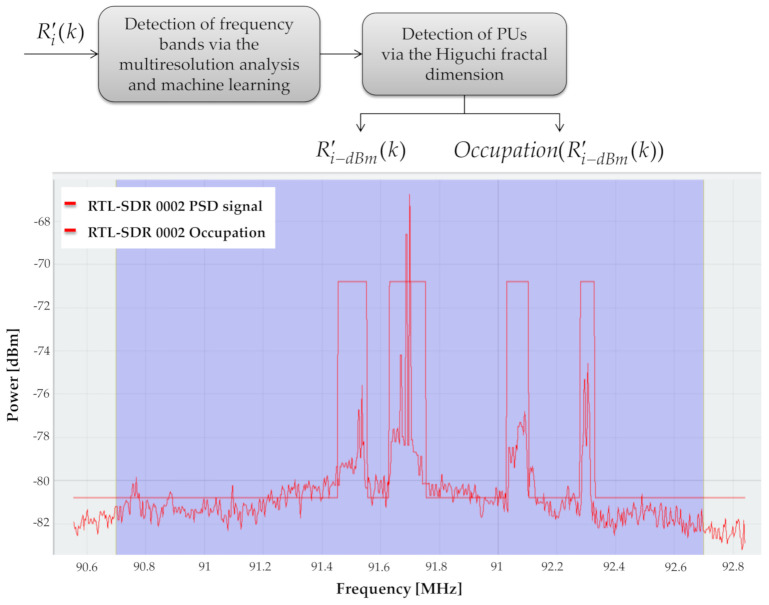
Delivered signals from the “detection of primary users” block.

This block consists of different submodules, as shown in Figure 14: (i) detection of frequency bands via the MRA and ML (in this submodule, the MRA is used to obtain the approximation coefficients, which are classified with the K-means algorithm and obtain the possible spectral windows; (ii) detection of PUs via the HFD (in this submodule, the autocorrelation of each window is calculated through the fractal dimension of Higuchi. At the end of this block, each spectral window is obtained on a logarithmic scale, as well as the occupation of each window. This MBSS technique was completely detailed in the previously published work by the authors in [12]. In this new proposal, the novelty is the developed platform to implement this technique using general-purpose communication devices.

In Figure 14, we can observe the result of applying this detection block. Here, the occupancy signal, indicating a *PU* transmission or noise, is displayed as a binary representation updated every 100 ms.

## 4. Real-Time Experiments and Results

This section presents the development environment, the parameters considered, and the results to validate the real-time MBSS using SDR devices through a controlled scenario.

### 4.1. Implemented Scenario

**Figure 15:**
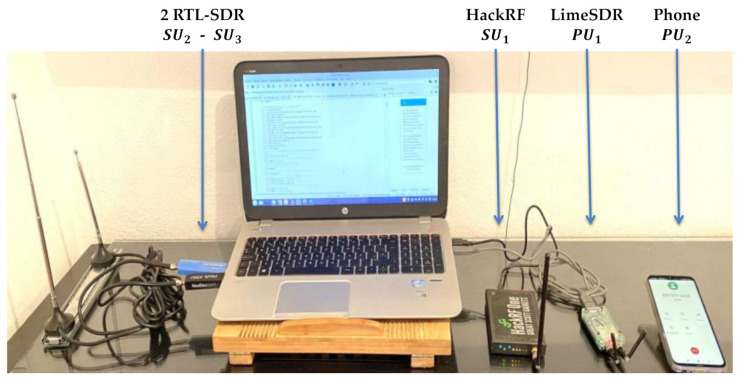
Implemented scenario.

The implemented environment is presented in Figure 15. Here, four SDR devices were employed. Three of these four SDR devices were established as *SU*_s_ and one was established as a *PU*. Additionally, a cellphone was used as a *PU*. Characteristics of each device used, as well as the parameters associated with the experimentation, are described in Table 2. In this real-time implementation, all these devices were controlled by an application developed by the authors that is briefly explained later.

**Figure 16:**
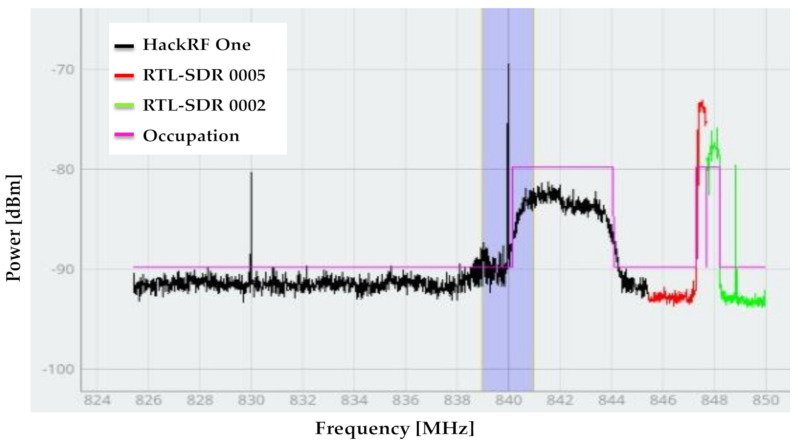
Frequency range perceived by *SU*_s_.

The order in which the SDR devices were located was dictated by the next priority: LimeSDR mini, followed by HackRF One and, finally, RTL-SDR. This priority was given by the bandwidth that each device can handle. Accordingly, the display and alignment of the signals in the application were carried out: (i) the display of the signals was based on the bandwidth of each connected device; (ii) the alignment of the signals was made according to the device that can perceive the widest bandwidth. That is, all the signals would be at the same power level as the device with the highest bandwidth; nevertheless, this processing does not change the shape of the PSD or the occupation of the signal in any way. This alignment only allows for better aesthetics in the application. In addition to the signal perceived by the (RTL-SDR), 1/8 of the beginning and 1/8 of the end of the frame were omitted, which means that 1/4 of the original frame was omitted (see Figure 14 and Figure 16). With this processing, distortion in the signal was avoided at the beginning and at the end. This distortion was due to the quality of the RTL hardware. Due to this phenomenon, the RTL-SDRs only perceived 2.4 MHz of bandwidth (see Table 2). Moreover, the sliding time window module had a delay (average) of 0.0065 s between the SDR devices and the PC (this value did not depend on the number of samples contained in the PSD). The PSD estimation module showed an execution time (mean) of 0.017 s in a frame with 512 samples, 0.018 s in a frame with 1024 samples, 0.02 s in a frame with 2048 samples, and 0.026 s in a frame with 4096 samples. The module for impulsive noise removal showed an execution time (average) of 0.00047 s in a frame with 512 samples, 0.00051 s in a frame with 1024 samples, 0.00071 s in a frame with 2048 samples, and 0.0011 s in a frame with 4096 samples. The estimation of frequency bands and detection of primary user module showed an execution time (mean) of 0.0339 s in a frame with 512 samples, 0.0468 s in a frame with samples, 0.0775 s in a frame with 2048 samples, and 0.1264 s in a frame with 4096 samples. This means that, on average, this implementation took 0.0621 s in a frame with 512 samples, 0.07181 s in a frame with 1024 samples, 0.10471 s in a frame with 2048 samples, and 0.156 s in a frame with 4096 samples. These values were measured using a laptop computer with 12 GB of RAM and an Intel i5 processor, and 10,000 frames were analyzed for each value.

According to the parameters and configurations mentioned in Table 2, the *SU*_s_ were observed as contiguous bands, as shown in Figure 16, which means that a total of 24.8 MHz of bandwidth was perceived. In this figure, it is shown that HackRF One observed the transmission made by $PU_{2}$, and the RTL-SDRs, $SU_{2}$, and $SU_{3}$ observed the transmission made by $PU_{1}$.

### 4.2. Signal Processing in the Controlled Implementation

**Figure 17:**
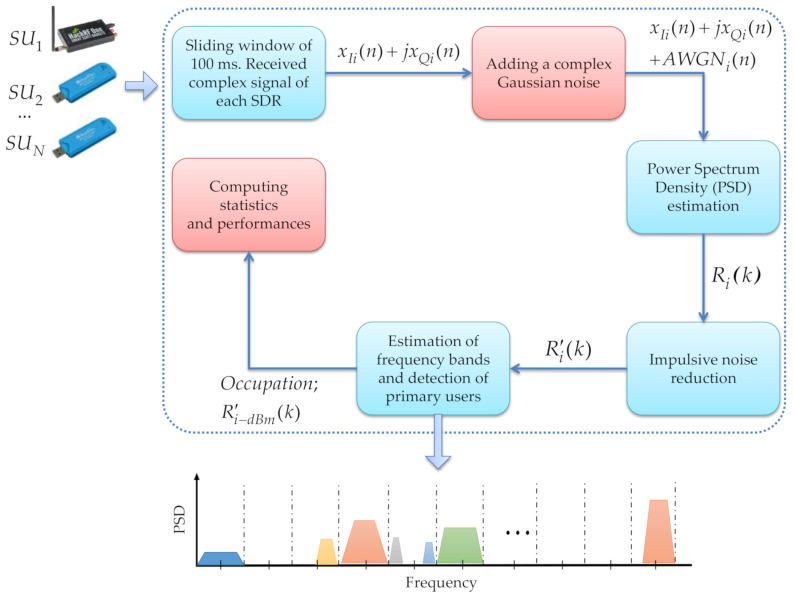
Controlled implementation steps.

This section describes in detail the processing and parameters used in the controlled implementation processing. Figure 17 shows two added modules (marked in pink color): (i) the first to add a complex Gaussian noise (AWGN, additive white Gaussian noise) noise in the time domain. This module permits modifying the SNR in the controlled implementation to have an environment to close to reality as much as possible. Noise is added to the signal perceived by the *SU*_s_ before processing it; (ii) the second is the block that stores the data and does the statistics to know the efficiency of this work in a real wireless communication environment.

**Figure 18:**
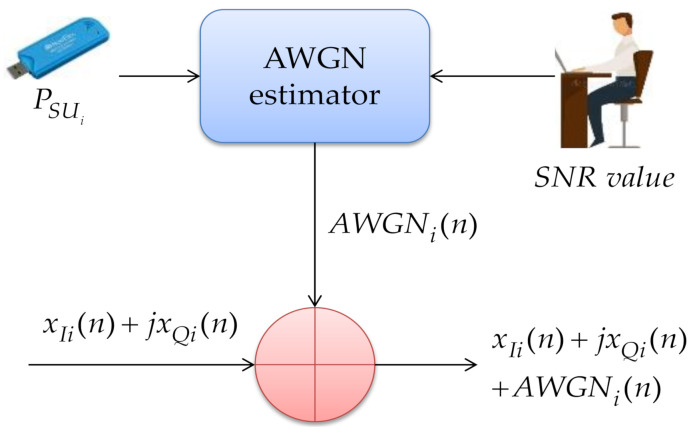
Block *Adding a complex Gaussian noise*.

Figure 18 shows the structure of the artificial noise addition block, where there are three input parameters: (i) the $P_{SUi}$ signal, which is the power with each device i transmits, (ii) the SNR value that the controlled experiment will have (this value is the same for each SDR device; see Table 3), and (iii) the complex signal $r_{i}(n)=x_{Ii}(n)+jx_{Qi}(n)$ perceived by each $SU_{i}$. The experimentation values for the controlled implementation are shown in Table 3. Each *SU* was sensed for 16.66 h at each SNR value, perceiving 2048 samples per frame for the HackRF One and 1024 samples per frame for the RTL-SDRs.

The artificial noise addition block uses the following pseudocode to calculate the noise that will be added to the $r_{i}(n)=x_{Ii}(n)+jx_{Qi}(n)$ signal:

- Function Artificial_Noise_Addition ($r_{i}(n)$, $P_{SUi}$, SNR_value)
-     Sigma = **float** ($P_{SUi}$/(10**(SNR_value/10)))
-     mu = 0
-     real = **np.random.randn** ((len(pxx)))*(sigma**0.5) + mu
-     imag = **np.random.randn** ((len(pxx)))*(sigma**0.5) + mu
- $\;\;AWGN_{i}(n)$ = real + j*imag
- $\;\;r_{i}(n)=r_{i}(n)+AWGN_{i}(n)$
- ***return*(**$r_{i}(n)$)

Lastly, the *Computing statistics and performance* block stores the $Occupation(R_{i-dBm}^{\prime }(k))$ signal that indirectly contains (i) the start and end edges of a transmission, which form the frequency windows, and (ii) whether these windows correspond to noise or a *PU* transmission. Subsequently, the signal transmitted by each $PU_{i}$ is compared to the signal received by each $SU_{i}$.

### 4.3. Results

**Figure 19:**
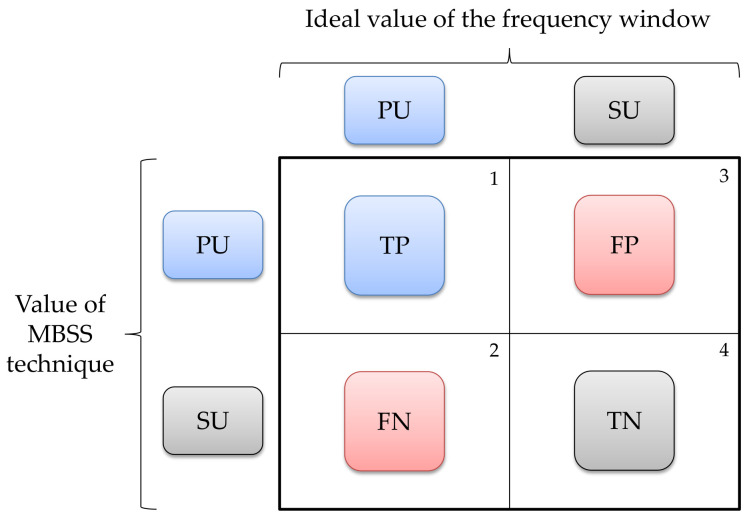
Classification of windows detected by the MBSS technique.

This section presents the results obtained for the MBSS technique. The two parameters evaluated in this implementation were, firstly, the probability of success (*PS*) which was the result of counting the total of correctly located frequency windows with respect to the total number of frequency windows. To determine this value, four possible cases were considered (Figure 19):

1. The window that corresponds to a *PU* transmission which *SU* classifies as *PU* transmission is considered a true positive (*TP*) value.
2. The frequency window that corresponds to a *PU* transmission which *SU* classifies as noise is considered a false negative (*FN*) value.
3. The window that corresponds to noise which *SU* classifies as a *PU* transmission is considered a false positive (*FP*) value.
4. The frequency window that corresponds to noise which *SU* classifies as noise is considered a true negative (*TN*) value.

As a function of these values, the *PS* is given by

(5) $$PS=\frac{TP+TN}{TP+FP+FN+TN}.$$

The second parameter is the number of samples in error, which is the number of samples between the edge of the *PU* transmission and the edge detected by the MBSS technique.

**Figure 20:**
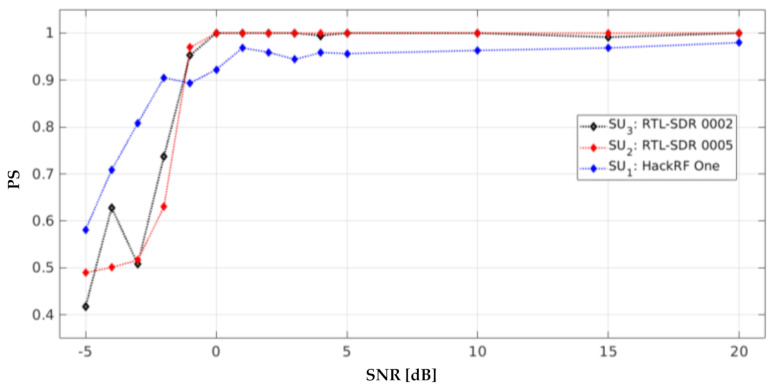
Probability of success of each *SU* in the controlled implementation.

Each SDR device used in this implementation had its own *PS* graph for each SNR value, as shown in Figure 20. The RTL-SDRs presented a better result to detect the frequency windows, which can be a *PU* transmission or noise, compared to the HackRF One. The *PS* for the RTL-SDRs was greater than 0.98 for values of SNR ≥ 0 dB and the *PS* for the HackRF One was greater than 0.94 for values of SNR > 0 dB. These values are very similar to those obtained in the simulated work [12] (i.e., *PS* greater than 0.98 for values of SNR > 0 dB).

**Figure 21:**
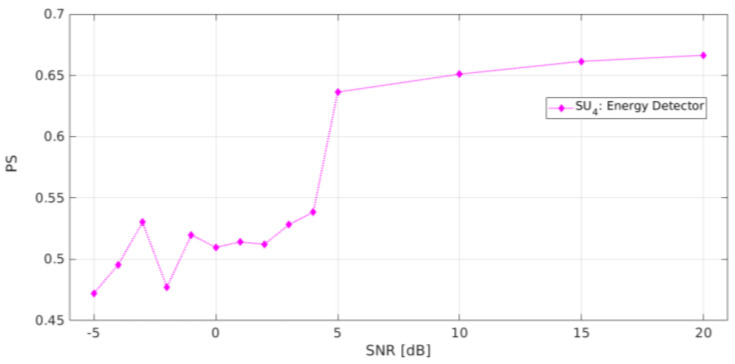
Probability of success of the energy detector.

Furthermore, the implementation of an energy detector (ED) [44] was carried out with an RTL-SDR (denoted $SU_{4}$) at $f_{C4}=846.2$ MHz with a bandwidth of 2.4 MHz, which means that $SU_{4}$ was perceived in the same radiofrequency space as $SU_{2}$. For this technique, the threshold of $SU_{4}$ was placed at −80 dBm, which is the same value that was chosen for the cluster selection stage in the algorithm to classify the approximation coefficients in [12] through the K-means technique. Figure 21 shows the result of applying this conventional ED. In this case, the average estimated *PS* was 0.64 for values of SNR ≥ 5 dB. This poor result is not surprising, due to the nature of the proposed methodology. Indeed, even if the considered band is a single one, the *PS* is calculated as an average of detected occupation windows inside the band (i.e., an MBSS is created for each considered SDR, thereby randomly varying the transmission location) to further decide if each detected window is free or not, via the HFD. In the case of the ED, many more windows are detected in error, inducing a lower *PS* estimate. This is due to the misclassification of the PSD signal by the ED in a short frequency range. This phenomenon greatly reduces the *PS* even though it correctly locates the *PU* transmission.

**Figure 22:**
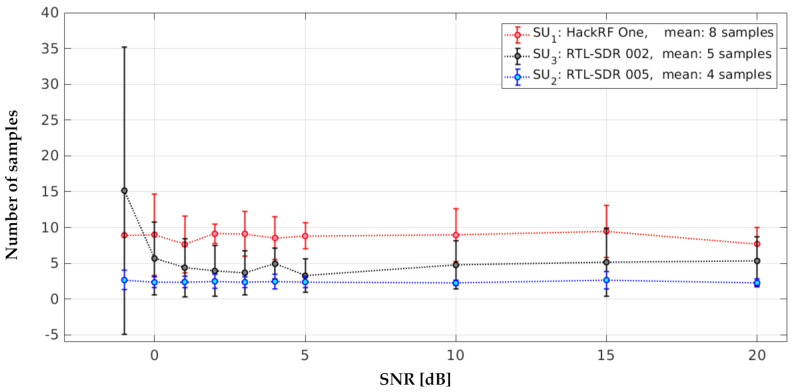
Samples in error for each *SU* in the implementation.

We also estimated the samples in error, defined as the samples that detected the start or end edge of a *PU* transmission. Figure 22 shows that, for values of SNR ≥ 0 dB, the samples in error were stable for each *SU*; however, the RTL-SDRs presented a better result for detecting *PU* with greater precision, with between three and five samples on average for samples in error, whereas the HackRF One had between eight and nine samples, which is a good performance. The samples in error presented in [12] were between two and three samples on average.

**Figure 23:**
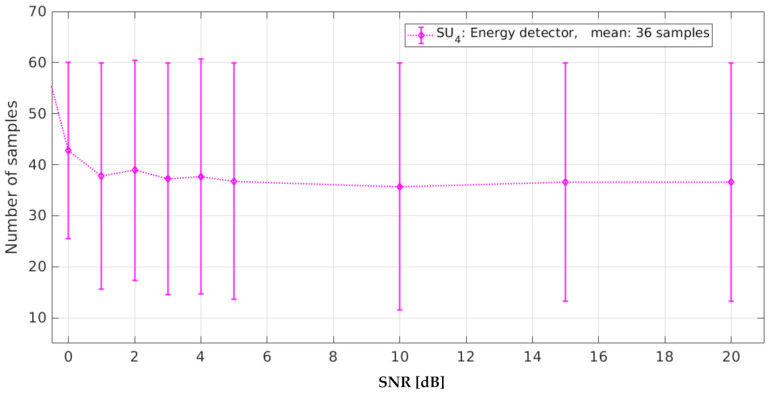
Samples in error for the energy detector.

The number of samples in error for $SU_{4}$ (conventional ED) was 36 on average (see Figure 23). Due to the nature of this methodology, events such as high-frequency noise, impulsive noise, or abrupt changes in the PSD generate more frequency windows due to the misclassification of the PSD signal by the ED in a short frequency range. ED stands out as a simple technique to implement with low processing resources. Nevertheless, it is a technique that tends to fail very quickly, especially when the environment has a lot of high-frequency noise or impulsive noise.

### 4.4. SDR–UAMI–MBSS Application

**Figure 24:**
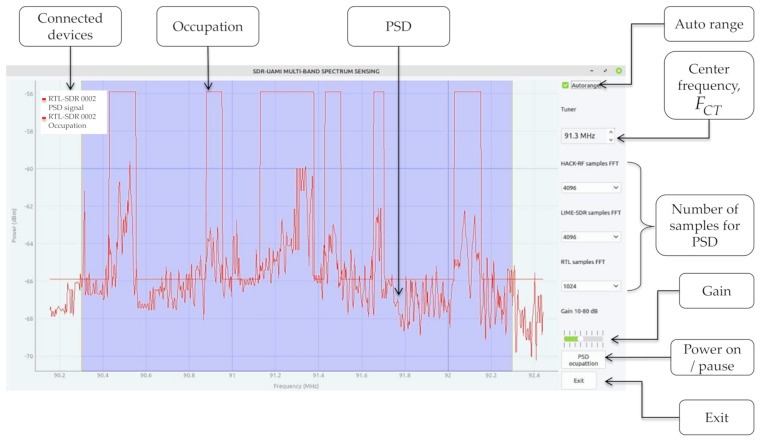
SDR–UAMI–MBSS application.

Lastly, the proposed methodology for the implementation of the MBSS technique using low-cost communication devices was consolidated in the development of an application named SDR–UAMI–MBSS. This interface shows the PSD and the occupation of a spectrum interval (see Figure 24), specifically, the radioelectric space that the *SU*_s_ (connected SDR devices) can visualize as a whole; it consists of advanced digital signal processing techniques and was developed in Python language. This application is easy to use, intuitive, and quite descriptive. This allows the user to have an easy interaction. Furthermore, it is an open-source application; it also allows knowing precisely what each module does, and whether it is possible to improve it. The SDR–UAMI–MBSS interface is a branch of the SDR–UAMI application [29].

## 5. Conclusions

In this work, an implementation for MBSS using SDR communication devices was presented. This development was done in Python language, and the algorithm works sequentially updating the information every 100 ms. The HackRF One device showed good performance in (i) correctly detecting noise or a possible *PU* transmission, and (ii) locating the start and end of a possible *PU* transmission. However, this performance cannot be significantly improved due to the noise cancellation module. Modifying the approximation coefficients for a neighboring colony indirectly affects the precision in detecting *PU* transmission. Practically, using this methodology, the precision for detection is sacrificed to improve the probability of success.

The impulse noise elimination module presented in this work showed good performance in eliminating high-frequency noise and abrupt changes in the signal. It was concluded that the implemented MBSS technique performed well, with similar results to the simulated results (0.98 of *PS* and two (mean) samples in error when locating the *PU* transmission) [12]. Nevertheless, this performance may be improved if this device uses a higher-gain antenna and if the SDR deployment is done in parallel, permitting the execution time to be reduced, as well as computing resources to be used more efficiently.

This MBSS technique also presented better performance than a conventional ED, in accurately detecting the transmission of the *PU* and in detecting its location. Moreover, with this algorithm, it is possible to use seven SDR devices seen as *SU*_s_. As future work, it is possible to develop a network for the MBSS using the same principles outlined in this work.

## Figures and Tables

**Table 1:** SDR device characteristics [31], (MSPS, mega samples per second).

Device	HackRF One	RTL-SDR	LimeSDR Mini
Frequency range	1 MHz–6 GHz	22 MHz–2.2 GHz	10 MHz–3.5 GHz
RF bandwidth	20 MHz	3.2 MHz	30.72 MHz
Sample depth	8 bit	8 bit	12 bit
Sample rate	20 MSPS	3.2 MSPS	30.72 MSPS
Tx channels	1	0	1
Rx channels	1	1	1
Duplex	Half	-	Full
Transmit power	−10 dBm + (15 dBm @ 2.4 GHz)	-	Max 10 dBm (depending on frequency)

**Table 2:** Parameters of the considered devices.

Tx/Rx	*SU*_1_	*SU*_2_	*SU*_3_	*PU*_1_	*PU*_2_
Device	HackRF One	RTL-SDR 0005	RTL-SDR 0002	LimeSDR Mini	Cell phone call
Tx Frequency (MHz)	-	-	-	847.8	842.5
Type of transmission	-	-	-	OFDM	CDMA [43]
Tx Bandwidth (MHz)	-	-	-	1	5
Rx Frequency (MHz)	835	846.2	848.6	-	-
Rx Bandwidth (MHz)	20	2.4	2.4	-	-

**Table 3:** Controlled implementation parameters.

	*SU*_1_	*SU*_2_	*SU*_3_
Device	HackRF One	RTL-SDR 0005	RTL-SDR 0002
SNR values	−5, −4, −2, −1, 0, 1, 2, 3, 4, 5, 6, 8, 10, 12, 14, 16, 18, and 20 dB		
Rx frames per SNR value	10,000	10,000	10,000
Samples per frame	2048	1024	1024

## Abbreviations

CR	Cognitive radio
*SU*	Secondary users
*PU*	Primary users
MBSS	Multiband spectrum sensing
SNR	Signal-to-noise ratio
IoT	Internet of things
ED	Energy detector
MRA	Multiresolution analysis
ML	Machine learning
HFD	Higuchi fractal dimension
SDR	Software-defined radio
USRP	Universal software radio peripheral
MIMO	Multiple-input multiple-output
Tx	Transmitters
Rx	Receivers
PSD	Power spectral density
MSPS	Mega samples per second
AWGN	Additive white Gaussian noise
*PS*	Probability of success
*TP*	True positive
*FN*	False negative
*FP*	False positive
*TN*	True negative
